# Deep Sequencing of Viroid-Derived Small RNAs from Grapevine Provides New Insights on the Role of RNA Silencing in Plant-Viroid Interaction

**DOI:** 10.1371/journal.pone.0007686

**Published:** 2009-11-05

**Authors:** Beatriz Navarro, Vitantonio Pantaleo, Andreas Gisel, Simon Moxon, Tamas Dalmay, György Bisztray, Francesco Di Serio, József Burgyán

**Affiliations:** 1 Istituto di Virologia Vegetale, Consiglio Nazionale delle Ricerche, Torino and Bari, Italy; 2 Istituto di Tecnologie Biomediche, Consiglio Nazionale delle Ricerche, Bari, Italy; 3 School of Computing Sciences, University of East Anglia, Norwich, United Kingdom; 4 School of Biological Sciences, University of East Anglia, Norwich, United Kingdom; 5 Corvinus University, Budapest, Hungary; Institute of Molecular and Cell Biology, Singapore

## Abstract

**Background:**

Viroids are circular, highly structured, non-protein-coding RNAs that, usurping cellular enzymes and escaping host defense mechanisms, are able to replicate and move through infected plants. Similarly to viruses, viroid infections are associated with the accumulation of viroid-derived 21–24 nt small RNAs (vd-sRNAs) with the typical features of the small interfering RNAs characteristic of RNA silencing, a sequence-specific mechanism involved in defense against invading nucleic acids and in regulation of gene expression in most eukaryotic organisms.

**Methodology/Principal Findings:**

To gain further insights on the genesis and possible role of vd-sRNAs in plant-viroid interaction, sRNAs isolated from *Vitis vinifera* infected by *Hop stunt viroid* (HSVd) and *Grapevine yellow speckle viroid 1* (GYSVd1) were sequenced by the high-throughput platform Solexa-Illumina, and the vd-sRNAs were analyzed. The large majority of HSVd- and GYSVd1-sRNAs derived from a few specific regions (hotspots) of the genomic (+) and (−) viroid RNAs, with a prevalence of those from the (−) strands of both viroids. When grouped according to their sizes, vd-sRNAs always assumed a distribution with prominent 21-, 22- and 24-nt peaks, which, interestingly, mapped at the same hotspots.

**Conclusions/Significance:**

These findings show that different Dicer-like enzymes (DCLs) target viroid RNAs, preferentially accessing to the same viroid domains. Interestingly, our results also suggest that viroid RNAs may interact with host enzymes involved in the RNA-directed DNA methylation pathway, indicating more complex scenarios than previously thought for both vd-sRNAs genesis and possible interference with host gene expression.

## Introduction

RNA silencing-based antiviral plant response is one of the best-studied antiviral strategies of plant. The key element of this strategy is the virus-derived small interfering RNA (vsiRNA), which guides RNA induced silencing complex (RISC) to target viral genomes in plants and invertebrates [1]. VsiRNAs are processed from double-stranded RNAs (dsRNAs) or structured single-stranded RNAs (ssRNAs) by RNase III-like enzymes such as DICER [2], [3] and, similarly to cell-derived small interfering RNAs (siRNAs), may guide the sequence-specific inactivation of target mRNAs by RISC [4].

Pathogenic RNAs like plant viruses are strong inducers as well as targets of RNA silencing and high levels of vsiRNAs accumulate during viral infection. However, despite extensive studies of siRNA biogenesis, the origin of vsiRNAs is still far from being fully understood. In virus infected plants, two distinct classes of vsiRNAs have been identified: the primary vsiRNAs, resulting from the cleavage of the initial trigger RNA by Dicer-like enzymes (DCLs)[5], and secondary vsiRNAs, whose biogenesis requires an RNA-dependent RNA polymerase (RDR) [6], [7]. The vsiRNAs are thought to be processed from viral dsRNA replicative intermediates, from local self-complementary regions of a viral genome or from dsRNAs resulting from the action of RDRs on viral RNA templates [1], [6].

DCL4 and DCL2 are the most important plant DICERs involved in ribovirus-induced RNA silencing and their products are vsiRNAs of 21 and 22 nt, respectively [8], [9]. However, in the case of nuclear-replicating begomoviruses with DNA genomes, DCL3 (which produces vsiRNAs of 24 nt) is likely involved in addition to DCL4 and DCL2 [6], [10]. Moreover, it has been shown recently that production of *Tobacco rattle virus* (TRV) derived vsiRNAs and antiviral silencing are strongly dependent on the combined activity of the host-encoded RDR1, RDR2, and RDR6 suggesting that they may convert viral ssRNAs into dsRNAs, which could serve as a substrate for vsiRNA production [11]. However, this model is not supported by previous observations indicating that the majority of vsiRNAs are derived from the plus (mRNA sense) viral strand [12], [13], [14]. A previous report also showed that RDR6 is not required for silencing the endogenous phytoene desaturase (PDS) gene using vectors based on the crucifer strain of tobacco mosaic virus (crTMV) and TRV [15]. These conflicting observations indicate that our knowledge about vsiRNA biogenesis is still limited, and that this process may depend on many factors including the replication strategy of the pathogen, the site of replication, and the nature of its genome.

The generated siRNAs associate with distinct Argonaute (AGO)-containing effector complexes to guide them to their RNA target molecules [1], [16], [17]. In plants, loading of siRNAs into a particular AGO complex is preferentially -but not exclusively- dictated by their 5′ terminal nucleotides [18]. AGO1 is presented as a major antiviral slicer, but other AGO paralogs are likely involved, potentially also mediating translational repression [19] or DNA methylation in a sequence-specific manner [6].

Viroids are the smallest plant infectious agents with a genome composed of a small non-protein-coding RNA that recruit host enzymatic machineries and redirect them to its replication and systemic movement (for a review see [20], [21], [22], [23], [24], [25]). Viroid species have been classified into the families *Pospiviroidae*, type species *Potato spindle tuber viroid* (PSTVd), clustering viroids localized in the nucleus wherein they replicate by an asymmetric rolling-circle mechanism [26], [27], and *Avsunviroidae*, type species *Avocado sunblotch viroid* (ASBVd), whose members accumulate and replicate in the chloroplast by a symmetric rolling circle mechanism wherein the cleavage steps are mediated by hammerhead ribozymes embedded in both (+) and (−) polarity strands [28], [29]. In the absence of coding properties, (+) polarity is assigned conventionally to the most abundant viroid strand accumulating *in vivo*. Consistent with the proposed replication mechanisms, both (+) and (−) monomeric circular genomic RNAs accumulate in tissues infected by members of the family *Avsunviroidae* [30], whereas only circular forms of (+) polarity are detected in tissues infected by members of the family *Pospiviroidae*, whose (−) RNAs accumulate at low levels and mainly as oligomeric intermediates in the replication pathway [31].

Viroid-specific highly-structured ssRNA and dsRNA species accumulate in infected plants [26], [32], suggesting that viroids similarly to plant viruses are potential activators of RNA silencing. Indeed, 21–24 nt viroid-derived small RNAs (vd-sRNAs) were identified in plant tissues infected by viroids of both families [33], [34], [35], [36], [37], [38]. These findings raised several still controversial questions on the role of RNA silencing in plant-viroid interaction (for reviews see [22], [23], [39]) including: i) whether viroid RNAs are both triggers and targets of this defence mechanism [40], [41], [42], [43], ii) if this is the case, how they may escape RNA silencing to systemically infect host plants, iii) whether vd-sRNAs are directly involved in viroid pathogenesis acting like microRNAs [33], [44] or trans-acting siRNAs [45], two special classes of cellular small RNA (sRNAs) targeting endogenous mRNAs (for a review see [46]), and iv) which viroid RNA(s) serve as template(s) for vd-sRNAs and in which subcellular compartment they are generated. With respect to the last question, (+) circular mature viroid RNAs have been proposed as the prevalent RNA substrates of the DCLs generating the vd-sRNAs from two nuclear replicating viroids [38], [40], but more complex scenarios can be also envisaged considering the nuclear co-localization of viroid replication and part of the RNA silencing machineries.

In the present study we analyzed the composition and the molecular nature of vd-sRNAs in grapevine infected by *Hop stunt viroid* (HSVd) and *Grapevine yellow speckle viroid 1* (GYSVd1), two members of the family *Pospiviroidae*, using the high-throughput Solexa sequencing platform to get a better insight into the biogenesis of vd-sRNAs. We identified 21-, 22- and 24-nt vd-sRNAs of (+) and (−) polarities. In contrast to previous observation using low scale sequencing, our results show a prevalence of (−) vd-sRNAs. We also show that the majority of vd-sRNAs emerge from very narrow hotspots of viroid genomes. Finally, our findings highlight new aspects of RNA silencing in the highly complex plant-viroid interactions.

## Results

### High-Throughput Sequencing Revealed Infection by Two Viroids

To establish the profile of the vd-sRNAs, four different cDNA libraries of sRNAs were generated from inflorescences (hereafter denoted flower), leaves, tendrils and berries of the Pinot noir grapevine clone ENTAV115. The cDNA libraries were sequenced on the Solexa high-throughput sequencing platform, yielding 3–6 million sequences for each library. Further processing of the raw deep sequencing data consisted of: i) removal of sequence tags with a non-matching 5′ or 3′-adapter or resulting from adapter self-ligation, ii) adapter trimming from the remaining tags, and iii) selection of sRNA sequences ranging in size between 16 and 26 nt. To improve the significance of comparisons among the four independent sequencing events, these data were normalized with respect to the 16–26 nt total sRNA reads from flower, the sample from which the most sRNAs were sequenced. Analysis of host-derived sRNAs will be described elsewhere.

The presence of vd-sRNAs in the populations of sequenced sRNAs was first revealed by searching for sRNAs perfectly matching the reference sequences of all known viroid species. The output of this first screening revealed the presence of sRNAs derived from HSVd and GYSVd1 in the grapevine libraries (data not shown), strongly suggesting that the tested plant was infected by these two viroids. Subsequent analyses of total RNA by RT-PCR, followed by cloning and sequencing of the amplification products, conclusively confirmed HSVd and GYSVd1 infections. We failed to detect *Citrus exocortis viroid* (CEVd), *Grapevine yellow speckle viroid 2* and *Australian grapevine viroid* (data not shown), which are the other viroids known to naturally infect grapevine [47]. Sequencing of several clones of HSVd and GYSVd1 full-length cDNAs showed limited sequence variability in the infecting viroid populations. These analyses identified the HSVd sequence variant already reported in databases with the accession number X06873 [48] as the most abundant (master sequence) in the infecting viroid population (Fig. S1). In contrast, a GYSVd1 master sequence was not identified because the sequenced variants of this viroid differed from each other at least in one position (Fig. S2). Sequence variability of the infecting viroid populations was taken into account to improve the search of vd-sRNAs (see below).

### HSVd- and GYSVd1-sRNAs Are Prevalently of (−) Polarity

**Figure 1:**
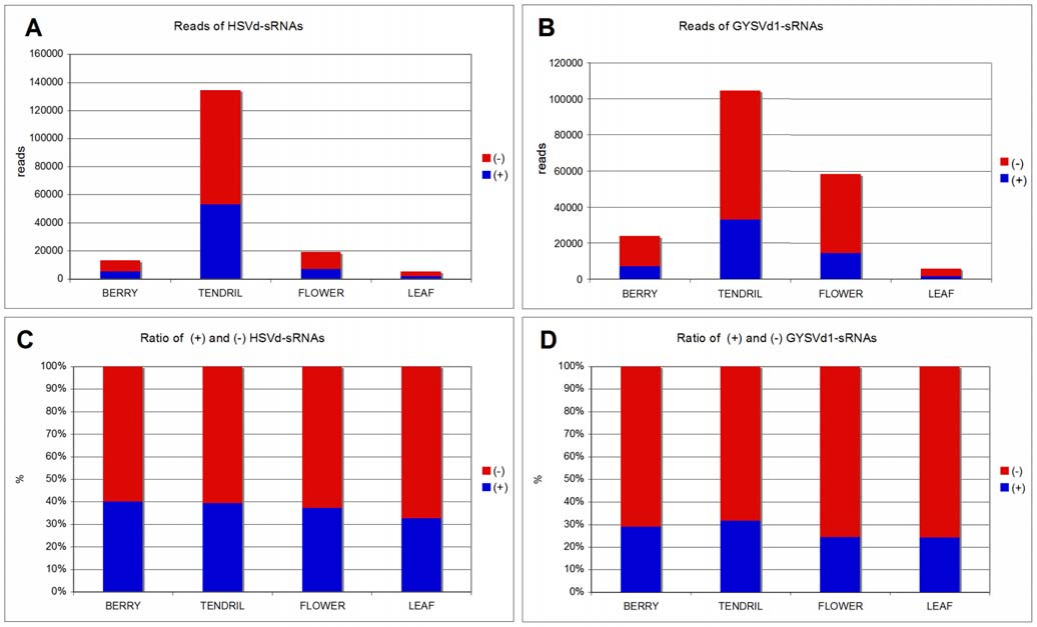
HSVd- and GYSVd1-sRNAs from grapevine tissues are prevalently of (−) polarity. Histograms comparing the total reads and the ratio of (+) and (−) HSVd-sRNAs (A and C) and GYSVd1-sRNAs (B and D) obtained by deep sequencing from berry, tendril, flower and leaf grapevine tissues.

To find as many vd-sRNAs as possible from the sequenced sRNAs populations, we searched for the sRNAs perfectly matching the sequence variants characterized here (Fig. S1 and S2) and all the sequence variants of HSVd and GYSVd1 previously reported from grapevine and deposited in databases. HSVd- and GYSVd1-sRNAs fulfilling these criteria were identified in all tissues, with the highest (about 135000 and 102500) and the lowest (about 5300 and 5800) reads resulting from tendril and leaf samples, respectively (Fig. 1A and 1B). Importantly, most of both HSVd- and GYSVd1-sRNAs were derived from their respective viroid (−) strand RNA: between 60 and 67% in HSVd-sRNAs, and between 70 and 75% in GYSVd1-sRNAs, with the highest (−)/(+) ratio being observed in leaf and flower samples in both cases (Fig. 1C and 1D). These findings, on the one hand, are in contrast with previous low-scale sequencing data from tomato infected by two members of the family *Pospiviroidae*, PSTVd [40] and CEVd [38], which revealed a prevalent accumulation of vd-sRNAs of (+) polarity. On the other hand, Machida et al. [37] identified similar levels of (+) and (−) PSTVd-sRNAs by low-scale sequencing in tomato infected by this viroid. In addition to a clear prevalence of (−) vd-sRNAs, our data also endow other questions related to their genesis and possible role in plant-viroid interaction (see below).

### HSVd- and GYSVd1-sRNAs Are Prevalently Composed of 21-, 22- and 24-nt Species

**Figure 2:**
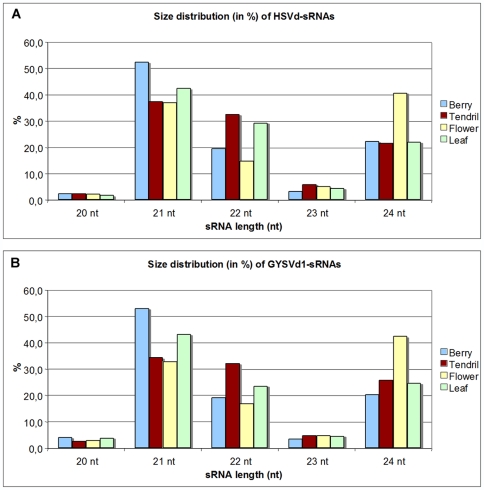
Size distribution of HSVd- and GYSVd1-sRNAs reveals prominent peaks of 21-, 22- and 24-nt species. Histograms comparing the size distribution of 20–24-nt HSVd-sRNAs (A) and GYSVd1-sRNAs (B) in the different grapevine tissues.

When the HSVd-sRNAs of 20–24 nt were grouped according to their size, distributions with prevalent peaks of 21-, 22- and 24-nt species were obtained (Fig. 2A), with those of 21 nt being the most abundant in berry, tendril and leaf samples (52%, 38% and 43% HSVd-sRNAs, respectively). By contrast, the most prominent peak from the flower sample corresponded to 24-nt HSVd-sRNAs (41%) prevailing over the 21-nt (37%) and almost doubling the 24-nt HSVd-sRNAs from the other tissues (Fig. 2A). Interestingly, the size distribution of GYSVd1-sRNAs from the four grapevine tissues was similar (Fig. 2B). The possibility that the prevalence of 24-nt vd-sRNAs in flowers could derive from a technical bias seems unlikely because, in the same experiments, the sequenced host-derived sRNAs mostly belong to the 21-nt size class in all tested tissues, including flower (data not shown).

These data point out that viroids infecting grapevine are targeted by DCLs generating different size classes of sRNAs, including the 24-nt sRNAs, and that this feature is not restricted to a single viroid species and/or to a single plant tissue. In line with the general major abundance reported above, (−) vd-sRNAs also prevailed in each size group and in all tissues (Fig. S3), indicating that they do not derive from a single DCL activity.

The relative abundance of 24-nt vd-sRNAs is again in contrast with previous results obtained by low-scale sequencing of vd-sRNAs from PSTVd and CEVd infecting tomato, in which two prominent peaks of 21- and 22-nt RNAs and negligeble [38], [40] or very low levels [37] of the 24-nt vd-sRNAs were detected. The tissues, developmental stages and different viroid-host combinations analyzed in each case could partially justify the divergent results reported above (see Discussion). In any case, the massive data generated by deep sequencing should allow to a more exhaustive retrieval than the limited datasets obtained by low-scale sequencing. Moreover, the existence in the infected tissues of vd-sRNAs of both polarity strands and ranging in size between 21- and 24-nt was confirmed by Northern-blot hybridization using leaf RNA preparations and digoxigenine-labeled full-length riboprobes for detecting HSVd (+) and (−) strands (data not shown).

### Uneven Distribution of the 5′ Nucleotide in Grapevine vd-sRNAs

**Figure 3:**
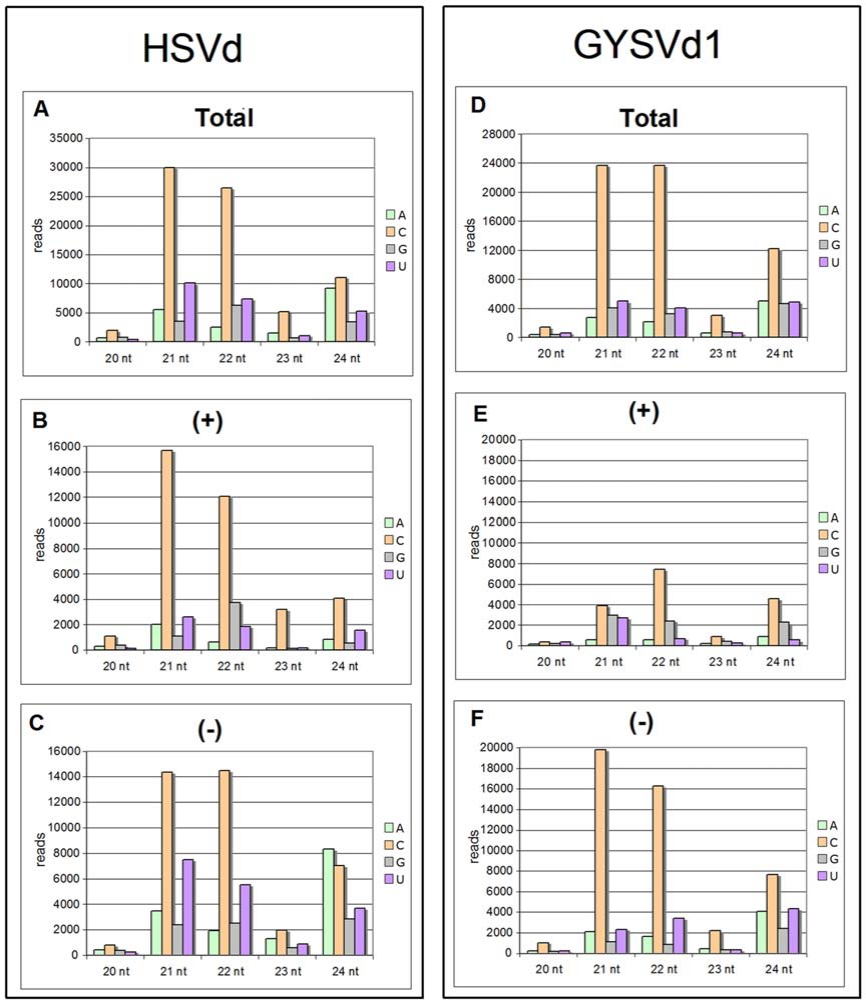
Relative abundance of vd-sRNAs with different size and 5′ termini. Histograms comparing the size distribution (20–24-nt) and nucleotide at the 5′ termini of total (A and D), (+) (B and E) and (−) (C and F) HSVd-sRNAs reads (left panels) and GYSVd1-sRNAs reads (right panels) from tendril.

Since the sorting process of sRNAs into effector Ago proteins is largely conditioned by their 5′-terminal nucleotide [18], we analyzed the nucleotide at this position in the grapevine vd-sRNAs. In HSVd, we observed the prevalence of C in all size groups and tissues (Fig. 3A and Fig. S4). U was the second most abundant residue at the 5′ terminal position in most cases except in 24-nt sRNAs, in which A was present with a frequency comparable to or higher than U (Fig. 3A and Fig. S4). When polarity of sRNAs was taken into consideration, C was confirmed as the most frequent residue at the 5′ terminal position of both (+) and (−) HSVd-sRNAs in all tissues and in most size classes (Fig. 3B and 3C, and Fig. S5), indicating that this feature is not dependent on the viroid RNA polarity. This analysis also showed that 24-nt HSVd-sRNAs with A residue at the 5′ terminal position were mostly of (−) polarity in all tissues including tendril, where they were prevalent among the (−) 24-nt HSVd-sRNAs (Fig. 3C and Fig. S5). Interestingly, similar results were obtained when GYSVd1-sRNAs were analyzed (Fig. 3D and Fig. S4) except that G was the second most frequent residue at the 5′ terminus of several (+) GYSVd1-sRNAs, including those from tendril (Fig. 3E and 3F, and Fig. S6). Altogether these data show that the frequency of specific residues at the 5′ terminal position of both (+) and (−) vd-sRNAs does not reflect the nucleotide frequency within their respective (+) and (−) viroid genomic RNAs and support the conclusion that the nucleotide at the 5′ terminal position of vd-sRNAs from both viroids is unevenly distributed. The high coincidence of the distribution profiles in all tissues and for both viroids is noteworthy, and corroborates the reproducibility of the results here obtained.

### Most HSVd- and GYSVd1-sRNAs Derive from Specific Regions of the Genomic (+) and (−) RNAs

**Figure 4:**
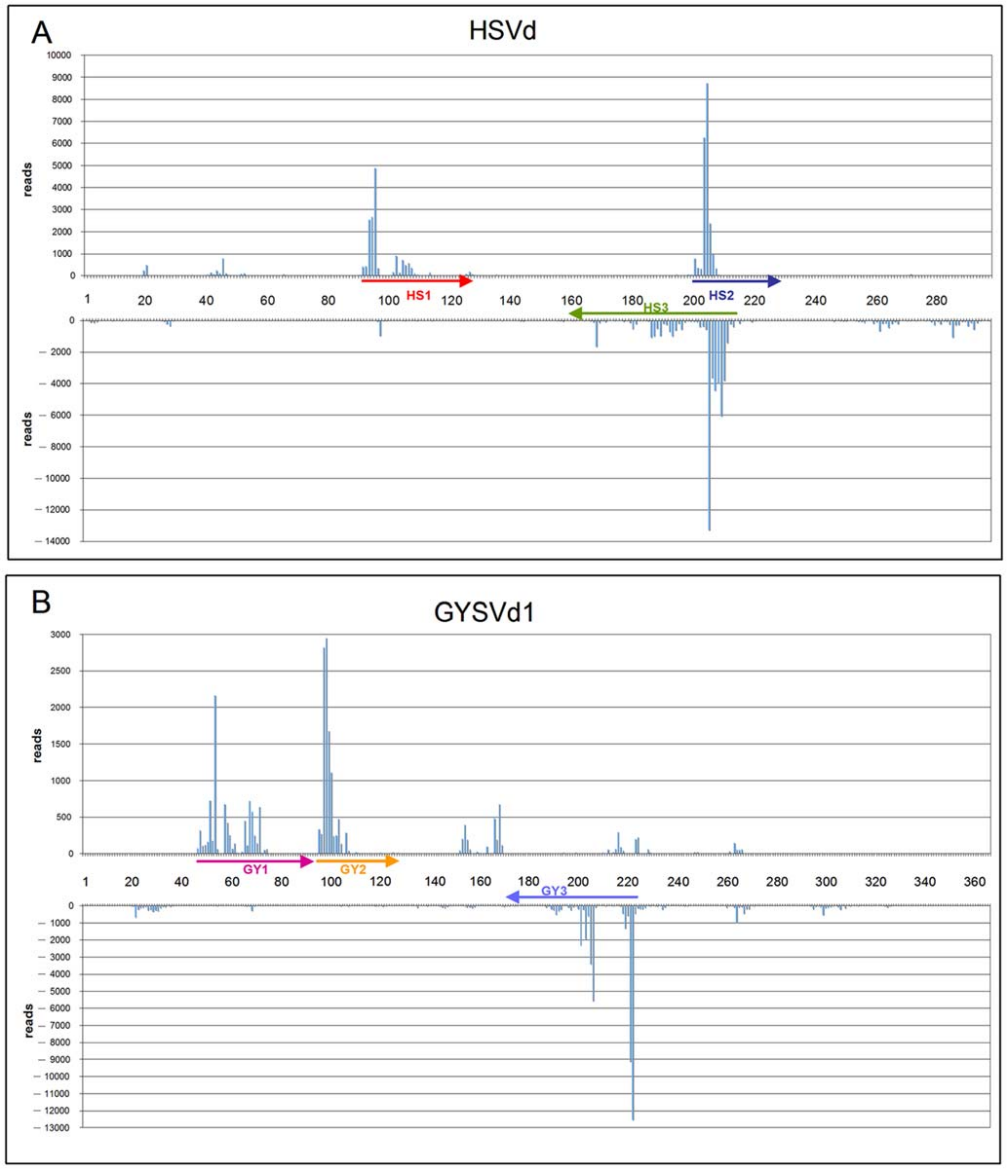
Most HSVd- and GYSVd1-sRNAs derive from restricted regions of the genomic (+) and (−) RNAs. Location of the 5′ termini and frequency of the HSVd-sRNAs (A) and GYSVd1-sRNAs (B) from tendril in their corresponding (+) and (−) genomic RNAs. Positives values correspond to vd-sRNAs of (+) polarity and negative values correspond to the vd-sRNAs of (−) polarity. Note that the scale is different in the panels and that the same numbers are used in the (+) polarity (5′→3′ orientation is from left to right) and in the (−) polarity (5′→3′ orientation is from right to left). For the location of the 5′ termini of vd-sRNAs we have considered the HSVd and GYSVd1 sequence variant with the accession numbers X06873 [48] and GQ995473, respectively. The viroid sequences covered by vd-sRNAs bellowing to hotspots (HS1, HS2 and HS3 for HSVd and GY1, GY2 and GY3 for GYSVd1) are denoted by arrows whose sense indicates 5′→3′ orientation.

**Figure 5:**
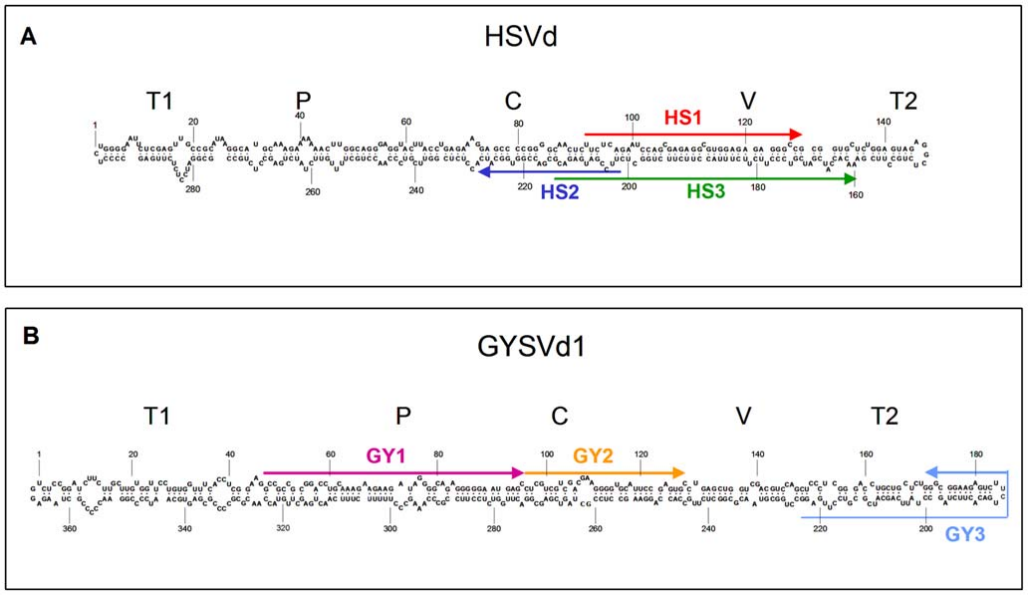
HSVd-sRNAs and GYSVd1-sRNAs do not cover the same viroid structural domains. Sequence and computer-predicted secondary structure for the (+) strand of the HSVd (sequence variant X06873) (A) and the GYSVd1 (sequence variant GQ995473) (B), corresponding to the master and the consensus variants in the grapevine sequenced viroid populations, respectively. The viroid sequences covered by vd-sRNAs corresponding to hotspots (HS1, HS2 and HS3 for HSVd, and GY1, GY2 and GY3 for GYSVd1) are denoted by arrows whose sense indicates 5′→3′ orientation. The position of five structural domains proposed for PSTVd and closely-related viroids [80] are indicated (P: pathogenic; V: variable; C: central; T1: terminal left; T2: terminal right), although no data on the functional properties of these regions in HSVd and GYSVd1 are available. The secondary structures were obtained by the program Mfold [81].

Non-redundant HSVd-sRNAs from grapevine tissues almost covered the whole viroid genome (data not shown). However, when the cloning frequency of each sRNAs was considered, their distribution profile showed that they mostly derive from specific and very restricted regions (hotspots) of the HSVd genomic (+) and (−) RNAs. Figure 4A illustrates HSVd-sRNA mapping from tendrils, but similar distribution profiles were obtained with HSVd-sRNAs from the other tissues (Fig. S7 and S8). Approximately 85% and 76% (+) and (−) HSVd-sRNAs mapped at only two and one major hotspots, respectively, hereafter denoted HS1, HS2 and HS3. The hotspots ranged in size between 30 and 50 nt and in total covered only 20% of the viroid genome. In the rod-like secondary structure proposed for HSVd, (+) sRNAs hotspots (HS1 and HS2) mapped to the upper and lower strands, partially covering the central and the variable domains (Fig. 5A). Although these two hotspots partially overlap in the viroid rod-like secondary structure, complementary vd-sRNAs with two 3′-protruding nucleotides in each strand were not found, suggesting that the (+) sRNAs from HS1 and HS2 were not concurrently generated by DCLs targeting the structured circular (+) genomic RNA. The (−) HSVd-sRNAs hotspot (HS3) mapped to the lower strand of the viroid rod-like secondary structure, partially overlapping with HS2 (Fig. 5A). Also in this case, complementary vd-sRNAs with two 3′-protruding nucleotides in each strand composed by (+) and (−) HSVd-sRNAs from the two overlapping hotspots were not identified, suggesting that these HSVd-sRNAs cannot be considered concurrent products of common DCL-mediated cleavages. HSVd-sRNAs of different sizes mapped to each hotspot (see below), suggesting that several DCLs accessed the same restricted RNA genomic regions. Taking advantage of the observation that the HS2 hotspot corresponds to a genomic sequence wherein a polymorphic position in the HSVd-infecting population was also mapped (Fig. S1), we could confirm that most vd-sRNAs (90%) from this region had a nucleotide composition corresponding to that of the identified HSVd master sequence (accession number: X06873), supporting the quantitative reproducibility of our data and their consistency with the genomic variability of the infecting HSVd population.

Mapping GYSVd1-sRNAs from tendrils gave similar results: most (+) sRNAs derived from only two hotspots (GY1 and GY2), whereas those of the (−) polarity mostly concentrated at a single hotspot (GY3) (Fig. 5B). Similarly to HSVd, 79% and 75% of (+) and (−) GYSVd1-sRNAs mapped to short regions covering only 22% and 17% of the genomic (+) and (−) viroid RNAs, respectively. Hotspots GY1 and GY2, containing (+) sRNAs, mapped to the left side of the central region and to the central region itself of the predicted GYSVd1 rod-like secondary structure, respectively, whereas hotspot GY3, containing (−) sRNAs, mapped to the terminal right domain including the terminal loop (Fig. 5B). Therefore, in contrast to HSVd, (+) and (−) GYSVd1-sRNAs mapped to distal and not overlapping regions, suggesting that localization of vd-sRNAs hotspots within the genome is an intrinsic property of the infecting viroid RNAs, thus differing between viroid species and between (+) and (−) strands of the same viroid RNA. Paralleling HSVd results, genome mapping of (+) and (−) GYSVd1-sRNAs from berry, flower and leaf tissues, generated distribution profiles essentially coincidental with those obtained from tendrils (Fig. S9 and S10). These data, besides confirming again the reproducibility of the deep sequencing method, also suggest that all tissue samples, including leaves from which the lowest level of vd-sRNAs was recovered, allowed exhaustive vd-sRNAs characterization.

**Figure 6:**
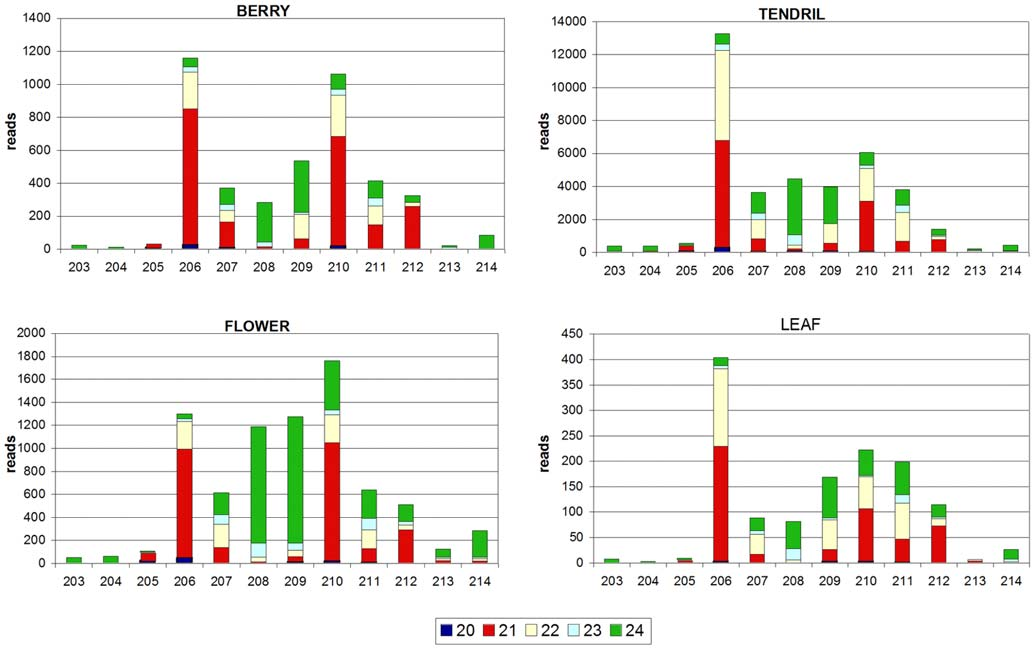
Specific size-classes vd-sRNAs may largely prevail at certain genomic positions. Histograms comparing location of the 5′ termini, frequency and size distribution of (−) vd-sRNAs corresponding to the HSVd hotspot 3 (HS3) and recovered from the different grapevine tissues. Numbers are referred to HSVd sequence variant with the accession number X06873. 5′→3′ orientation is from right to left.

A further size distribution analysis within the hotspots showed that in most cases the 5′ terminus of vd-sRNAs of different sizes mapped concurrently to the same position, although specific size-class sRNAs may largely prevail at certain genomic positions, as exemplified by Figure 6 showing details of HS3. In this case, positions 206, 210 and 212 correspond prevalently to the 5′ termini of 21-nt sRNAs, whereas positions 208, 209 and 214 were almost exclusively occupied by the 5′ termini of 24-nt sRNAs (Fig. 6). We do not know whether the differential sRNA distribution within the hotspots may have any significance, although the highly coincidental profiles of the four independent sequencing experiments are intriguing. Similar considerations can be extended to the other vd-sRNAs hotspots (data not shown).

Mapping data of both HSVd- and GYSVd1-sRNAs were validated by Northern-blot hybridization using probes consisting of DNA oligonucleotides targeted against viroid genomic regions characterized by high (hotspots) and low vd-sRNA densities (Fig. 5). The obtained hybridization signals with hotspot-specific probes were much stronger than those generated by probes specific for viroid regions with low density of vd-sRNAs (data not shown), thus supporting the reliability of the deep sequencing data.

## Discussion

In this work, high-throughput sequencing of sRNAs from different grapevine tissues has been applied to further characterize the vd-sRNAs of two nuclear-replicating viroids, HSVd and GYSVd1, and new data about their genesis and possible biological roles have been obtained. Previous efforts of characterizing vd-sRNAs by low-scale sequencing revealed that (+) vd-sRNAs of PSTVd [40] and CEVd [38] are the most abundant in tomato plants infected by these viroids. Based on these findings, it was proposed that vd-sRNAs mostly derive from direct DCL targeting of (+) viroid genomic RNA, likely its circular form, which is the most abundant viroid RNA accumulating *in vivo*. In contrast, our data (Fig. 1 and Fig. S3) show that (−) vd-sRNAs largely dominate the sRNA populations of both HSVd and GYSVd1 in grapevine, strongly supporting that a more complex mechanism is involved in vd-sRNAs biogenesis. This consideration is in line with a similar prediction by Machida et al. [37], who identified relatively abundant (−) PSTVd-sRNAs in tomato leaves by low-scale sequencing. Whether the prevalent presence of (−)-vd-sRNAs as reported here is applicable to other viroids remains to be seen by deep sequencing vd-sRNAs in other viroid-host combinations. The (−) vd-sRNAs could derive from DCL(s) targeting dsRNAs generated during the nuclear replication or resulting from the activity of host RDRs recognizing some viroid RNA features (i.e. the lack of cap structure and poly-A tail) as aberrant traits [49], [50]. Alternatively, DCL enzymes may preferentially target the (−) multimeric replicative intermediates, which are highly-structured ssRNAs. It is worth noting that RNA samples used in the present study were from a grapevine plant grown in the field. This plant was obtained by vegetative propagation from a mother plant presumably already infected by both HSVd and GYSVd1, which are the most widespread viroids in grapevine. Therefore, infection of both viroids in this woody plant, which did not show any symptoms, was very likely in stationary stage. We can assume that some of the above-mentioned studies with herbaceous host like tomato, performed at a logarithmic-stage of viroid infection, may show different vd-sRNA profiles, since the accessibility to the RNA silencing machinery of different viroid RNA species may be continuously changing. In line with this hypothesis, the results of Machida et al. [37] showed that the distribution profiles of PSTVd-sRNAs from symptomatic tomato leaves became more heterogeneous with time.

Assuming that the four DCLs identified in the grapevine genome [51], [52] have the same subcellular localization and biochemical activities as their homologous in *Arabidopsis* [53], the prevalence of 21-, 22- and 24-nt species observed in the size distribution profiles of the sequenced HSVd- and GYSVd1-sRNAs (Fig. 2 and Fig. S3) indicates that viroid RNAs are targeted by multiple DCLs. The 21-nt vd-sRNAs could derive from the nuclear activity of DCL1 targeting highly-structured genomic viroid RNAs of both polarity strands by a mechanism resembling miRNA biogenesis and/or from the activity of DCL4, which acts in concert with RDR6 in *Arabidopsis* [54]. Instead, the 22-nt vd-sRNAs likely derive from the activity of DCL2 that is hierarchically involved in antiviral defense together with DCL4 [8], [9], [55], [56].

Identification of 24-nt vd-sRNAs in grapevine is consistent with the prediction that viroids are also targeted by a DCL homologous to the *Arabidopsis* DCL3 [51], [52]. Because 24-nt sRNAs are involved in RNA directed-DNA methylation (RdDM), our finding opens new scenarios on both the biogenesis and possible role of vd-sRNAs in plant-viroid interaction. DCL3 acts in concert with ARGONAUTE 4 (AGO4), RDR2, and RNA polymerases IV and V (Pol IV and V) in a spatio-temporal regulated pathway. Current models propose that 24-nt sRNAs are loaded into AGO4 for targeting and methylating DNA by *de novo* cytosine methyltransferase, DRM2, in concert with Pol V and other proteins [57], [58]. Due to their nuclear replication and localization, certain viroid RNAs could enter this pathway at different levels. Qi and Ding (2003) showed that both (+) and (−) PSTVd RNAs localize in the nucleoplasm, whereas the (+) polarity strands prevalently accumulate in the nucleolus, wherein presumably they are cleaved and circularized [59]. However, it is unlikely that the highly-structured genomic (+) viroid RNA accumulating in the nucleolus is directly targeted by DCL3 because this scenario does not explain why both (+) and (−) 24-nt vd-sRNAs have been identified in infected grapevine tissues (Fig. S3). A more likely alternative is that (+) viroid RNAs migrating to the nucleolus could be recognized by RDR2, thus entering the DCL3 degradation pathway at this level.

Identification of high levels of 24-nt vd-sRNAs in infected grapevine is consistent with the previous finding that replicating viroid RNAs induce *de novo* methylation of homologous transgenic DNA sequences, the first proof that DNA methylation is an RNA-mediated process [60]. Whether viroids may directly or indirectly interfere with host DNA methylation profiles, as well as whether 24-nt vd-sRNAs may target DNA methylation of host genes, is not known and remains an interesting challenge for next studies on viroid pathogenesis. However, it is noteworthy that the activity of an RNase-III like enzyme has been involved in the cleavage step of (+) viroid multimeric forms during replication of nuclear viroids [61], suggesting that interaction between viroid RNAs and RNA silencing pathways could be more complex than suspected before. In line with this considerations, it is important that a peak corresponding to 24-nt vd-sRNAs has not been identified in the size distribution profiles of vd-sRNAs from the chloroplast-replicating *Peach latent mosaic viroid* sequenced by low-scale [62] and deep sequencing [63]. Altogether these data support the notion that vd-sRNAs of members belonging to the families *Pospiviroidae* and *Avsunviroidae* arise, at least in part, from different pathways as a result of their diverse subcellular replication sites. In this same context, targeting of nuclear-replicating viroid RNAs by DCL3 supports that some vd-sRNAs are indeed generated in the nucleus. Pertinent in this respect is the observation that DCL2 and DCL4 can process Pol-IV derived dsRNAs when DCL3 is mutated in Arabidopsis [64], which suggests the possibility that 21- and 22-nt vd-sRNAs of nuclear replicating viroids could partially derive from a similar redundant activity of host DCLs. Further studies are needed to establish whether the 21- and 22-nt vd-sRNAs are actually produced in the nucleus before being exported to the cytoplasm wherein vd-sRNAs seem to accumulate [65], or directly in the cytoplasm or in both subcellular compartments. Thus, the possibility exists that the RNA silencing machinery targets nuclear viroids at several key points of their infectious cycle, including replication and cytoplasmic trafficking [66].

Mapping of vd-sRNAs to the viroid genomic RNAs has provided several insights on their genesis and biological function. The few number of hotspots in both (+) and (−) viroid genomic RNAs (Fig. 4), on the one hand, suggests that very limited regions of the viroid genome are potentially targeted by DCLs and, on the other hand, reinforces the view that the (+) circular forms cannot be the prevalent substrate for DCL-mediated generation of grapevine vd-sRNAs, as suggested before in other viroid-host combinations [38], [40]. Therefore, besides the genomic (+) and (−) viroid RNAs, the viroid-derived dsRNAs synthesized during replication or by host RDRs could be proper substrates for generating both (+) and (−) vd-sRNAs. However, the distribution profiles of vd-sRNAs do not support the possibility that vd-sRNAs mapping to (+) and (−) hotspots derive directly from the same dsRNA molecule because they do not form duplexes with two 3′-protruding nucleotides, the hallmarks of DCL activity. Therefore, (+) and (−) vd-sRNAs appear to be generated by two independent processes, additionally suggesting that the limited accessibility to DCLs is an intrinsic property of each viroid RNA. In line with this view, the hotspots of HSVd-sRNAs and GYSVd1-sRNAs do not cover the same viroid structural domains (Fig. 5). However, it is intriguing that, for each viroid strand, the vd-sRNAs of all size classes actually map to the same regions, indicating similar preferences of the different DCLs.

Possible explanations for the hotspot profiles of vd-sRNAs from both viroids, which have been confirmed by Northern-blot hybridization, include: i) certain RNA-binding proteins [67] may protect genomic regions of (+) and (−) viroid RNAs from DCL digestion; ii) RDR(s) could have low processivity and synthesize short viroid dsRNAs; iii) vd-sRNAs might be differentially targeted by one or more exoribonucleases, like those acting upon mature miRNAs in Arabidopsis [68]; and iv) vd-sRNAs could have differential stability depending on whether they are or not incorporated into RISC complexes containing distinct AGO members [69]. In this respect, it is noteworthy that the 5′ terminal nucleotide of most grapevine vd-sRNAs is C residue (Fig. 3). Sorting of Arabidopsis sRNAs is largely directed by the 5′ terminal nucleotide [18] and sRNAs with a C at this position are preferentially recruited by AGO5, whose function has not been explored. Moreover, the second most abundant nucleotide at the 5′ terminal position of grapevine 24-nt vd-sRNAs (the A residue) is preferentially found at the same position in the 24-nt sRNAs recruited by Arabidopsis AGO4, which acts in concert with DCL3, suggesting that 24-nt vd-sRNAs may enter the DNA methylation pathway.

It should be mentioned that our protocol used for the preparation of sRNAs cDNA library is not apropriate to amplify and sequence sRNAs that are the products of unprimed RNA synthesis catalyzed by host RDR [70]. Since they are product of RNA synthesis they have 5′-triphosphate, which does not allow the ligation of the 5′-adaptor to sRNAs. Thus, if these sRNAs exist, they escaped from our analysis, although the existence of this type of sRNAs has not been reported from plants. In addition, very recent analysis of tombusvirus derived vsiRNAs failed to detect sRNAs produced by unprimed RDR synthesis (Szittya et al., unpublished).

Viroid-derived small RNAs show remarkable similarites to as well as differences from plant virus-derived small RNAs. Plant viruses with RNA genome replicate in cytoplasm and generate predominantly 21- and 22-nt long vsiRNA [11], [13], [71], [72], while the vsiRNAs deriving from plant DNA viruses replicating in the nucleus are mostly 21-, 22- and 24-nt long [73]. Interestingly, in contrast to a chloroplast replicating viroid [62], [63], two members of nuclear-replicating viroids also generate vd-sRNAs of 21-, 22- and 24-nt, suggesting that the site of replication of invading nucleic acids is an important factor in the genesis of their small RNAs. Although both viruses and viroids show uneven sRNAs distribution profiles along their respective genomes, the prevalent polarity of sRNAs varies remarkably depending on the specific system, the replication strategy and, very likely, the infection stage.

Finally we would like to remark on the high potential offered by grapevine for studying plant-viroid interactions. For example, taking advantage of the available complete grapevine genome sequence, we searched for possible grapevine targets of the sequenced vd-sRNAs and identified only one perfectly maching 21-nt HSVd species, whereas several additional targets were identified with one or two mismatches. However, the frequency of vd-sRNAs having a putative target in grapevine genome was always extremely low and information on possible functional roles of these potential genomic targets are still lacking. Therefore, to understand whether vd-sRNAs may actually have any direct biological impact on host gene expression could be an interesting aim for future studies.

## Materials and Methods

### Plant Material

All plant material was from *Vitis vinifera*, cultivar “Pinot noir”, clone ENTAV115 grown in collection fields. Young leaves and tendrils were collected from 1st to the 3th internode from the shoot apex. The inflorescences were collected at their appearance, whereas the small fruits used were 1–4 mm in size.

### Extraction, Fractionation and Sequencing of Grapevine sRNAs

*Vitis vinifera* total RNA was extracted following the method reported by Turturo et al. [74] except for the silica particle absorption. Low molecular weight RNA (LMWR) was further enriched by using Quiagen RNA/DNA midi kit and following the manual procedures. LMWR was used to generate short RNA libraries as indicated by German et al. [75]. Deep-sequencing was done on Illumina Solexa platform using the standard protocol of manufacturer.

### Characterization of Infecting Viroids

Total RNA preparations were obtained from grapevine leaves as reported by Turturo et al. [74]. cDNAs were synthesized by using the high-capacity cDNA Reverse Transcription kit as suggested by the supplyer (Applied Biosystems). PCR amplification reactions were carried out with the following primer pairs: HSVd-83M-Rev: 5′-AACCCGGGGCTCCTTTCTCA-3′ and HSVd-78P-For: 5′-AACCCGGGGCAACTCTTCTC-3′, specific for full-length HSVd cDNA amplification [76]; GYSVd1-Rev: 5′-GCGGGGGTTCCGGGGATTGC-3′ and GYSVd1-For: 5′-TAAGAGGTCTCCGGATCTTCTTGC-3′, specific for full-length GYSVd1 cDNA amplification; GYSVd2-C2-Rev: 5′-CCGAGGTGTAACCACAGGGAACC-3′ and GYSVd2-H1-For: 5′-TTGAGGCCCGGCGAAACGC-3′, specific for partial (194 bp) GYSVd2 cDNA amplification [74]; CEVd-C1Rev: 5′-CGAAAGGAAGGAGACGAGCTCCTG-3′ and CEVd-H3For: 5′-TTCAGGGATCCCCGGGGAA-3′, specific for partial (115 bp) CEVd cDNA amplification [77]; AGVd-Rev: 5′-CTCGACGACGAGTCGCCAGGTGAG-3′ and AGVd-For: 5′-GTCGACGAAGGGTCCTCAGCAGAG-3′, specific for full-length (375 bp) AGVd cDNA amplification. Full-lenght HSVd and GYSVd1 monomeric cDNAs were cloned in p-GEM-T- easy vector (Promega) and sequenced. Multiple alignments of viroid sequence variants were performed with the CLUSTAL W program [78].

### Sequence Analysis of HSVd- and GYSVd1-sRNAs

After trimming the adapters from the resulting sequences, they were sorted into separate files according to the length. Sequences between 20 and 24 nt were pooled and each set of sequences was analyzed by BLAST [79] against the nucleotide sequence of HSVd and GYSVd1 variants cloned and sequenced in this study and of variants of these viroid species previously reported on grapevine and deposited in databases (Materials and Methods S1). No mismatch was allowed and the circularity of the viroid genome was taken into consideration. A set of perl scripts to analyze and visualize the mapping data search for hot spots was developed.

### Northern-Blot Hybridization of vd-sRNAs with Oligodeoxyribonucleotides

LMWR were electrotransferred and fixed by UV irradiation to nylon membranes (Hybond-N, Amersham). The membranes were hybridized at 42°C in DIG-hybridization buffer (Roche) with riboprobes corresponding to the full length HSVd genome RNAs of both polarities. In additional experiments the membranes prepared as reported were hybridized at 37°C in Perfect-Hyb buffer (Sigma) with each of the following 5′-radiolabeled probes: Phs-1 (5′-GATGCCACCGGTCGCGTCTCATCGGAAG-3′) and Phs-2 (5′-CTTCTTTACCTTCTTCTGGCTCTTCCGATGAGACG-3′) complementary and identical to positions 201–229 and 180–214, respectively, and Phs-3 (5′-CAAAAGCAGGTTGGGACGAACCGAGAGGTGATGCC-3′) and Phs-4 (5′-GGCATCACCTCTCGGTTCGTCCCAACCTGCTTTTG-3′) complementary and identical to positions 223–257, respectively, of the HSVd variant X06873; Pgy-5 (5′-GCACTCGGAATGCACCCCTTCGTCGACGACGAG-3′) and Pgy-6 (5′-GCCTATTCAGCATCGCGTCCTTGAGGC-3′) complementary and identical to positions 96–128 and 198–224, respectively, and Pgy-7 (5′-GAGCTTGTACCAACGCGCCCCGCGAGTGCAATC-3′) and Pgy-8 (5′-GATTGCACTCGCGGGGCGCGTTGGTACAAGCTC-3′) identical and complementary to positions 315–346, respectively, of the GYSVd1 variant GQ995473. After overnight hybridization, the membranes were washed twice with 2X SSC plus 0.1% SDS for 10 min at room temperature, and once with 0.1X SSC plus 0.1% SDS at 55°C for 15 min, and examined with a bioimage analyzer (Fujifilm FLA-5100).

## Supporting Information

Figure S1 Multiple sequence alignment of HSVd cDNA variants from grapevine Pinot noir ENTAV115 identified in this study. Dashes and stars denote gaps and nucleotide identity, respectively. HSVd variants PN.1, PN.3, PN.7, PN.8, PN.10, PN.13 and PN.14 are identical to HSVd variant with accession number X06873, which is the master sequence in the infecting viroid population. Accession numbers for variants PN.9, PN.11 and PN.12 are GQ995464, GQ995465 and GQ995466, respectively. Nucleotides in red correspond to changes with respect to the master sequence. Numbers at the end of each line indicate nucleotide positions of each variant in the multiple alignment.
(1.69 MB TIF)

Figure S2 Multiple sequence alignment of GYSVd1 cDNA variants from grapevine Pinot noir clone ENTAV115 identified in the present study. Dashes and stars denote gaps and nucleotide identity, respectively. Nucleotides in red correspond to changes with respect to the consensus sequence, which corresponds to the sequence variant GYSVd1.PN.22 (accession number GQ995473) in the alignment. Numbers at the end of each line indicate nucleotide positions of each variant in the multiple alignment.
(1.16 MB TIF)

Figure S3 Size distribution (20–24 nt) of vd-sRNAs from different grapevine tissues. Histograms comparing the size distribution (20–24 nt) of HSVd- (upper panels) and GYSVd1-sRNAs (lower panels) isolated from berry (A and E), tendril (B and F), flower (C and G) and leaf (D and H). Vd-sRNAs of 21 nt of both viroids were the most abundant in berry, tendril and leaf samples, whereas the most prominent peak from the flower samples corresponded to 24-nt HSVd-sRNAs and GYSVd1-sRNAs.
(8.98 MB TIF)

Figure S4 Frequency of the 5′-terminal nucleotide in vd-sRNAs. Histograms comparing the size distribution (20–24-nt) and nucleotide at the 5′ termini of HSVd-sRNAs (upper panels) and GYSVd1-sRNAs (lower panels) from berry (A and D), flower (B and E) and leaf (C and F).
(10.22 MB TIF)

Figure S5 Frequency of the 5′-terminal nucleotide in (+) and (−) HSVd-sRNAs. Histograms comparing the size distribution (20–24-nt) and nucleotide at 5′ termini of (+) (left panels) and (−) (right panels) HSVd-sRNAs from berry (A and B), flower (C and D) and leaf (E and F).
(9.92 MB TIF)

Figure S6 Frequency of the 5′-terminal nucleotide in (+) and (−) GYSVd1-sRNAs. Histograms comparing the size distribution (20–24-nt) and nucleotide at 5′ termini of (+) (left panels) and (−) (right panels) GYSVd1-sRNAs from berry (A and B), flower (C and D) and leaf (E and F).
(9.65 MB TIF)

Figure S7 Mapping of the 5′ termini and frequency of (+) HSVd-sRNAs from different tissues. Berry (A), tendril (B), flower (C) and leaf (D). Note that the scale is different in the four panels and that 5′-3′ orientation is from left to right. Mapping is referred to the HSVd (+) genomic RNA (sequence variant with the accession number X06873).
(9.65 MB TIF)

Figure S8 Mapping of the 5′ termini and frequency of the (−) HSVd-sRNAs from different tissues. Berry (A) tendril (B), flower (C) and leaf (D). Note that the scale is different in the four panels and that 5′-3′ orientation is from right to left. Mapping is referred to the HSVd (−) genomic RNA (sequence variant with the accession number X06873).
(9.71 MB TIF)

Figure S9 Mapping of the 5′ termini and frequency of the (+) GYSVd1-sRNAs from different tissues. Berry (A) tendril (B), flower (C) and leaf (D). Note that the scale is different in the four panels and that 5′-3′ orientation is from left to right. Mapping is referred to the GYSVd1 (+) genomic RNA (sequence variant GYSVd1.PN.22 with the accession number GQ995473).
(7.56 MB TIF)

Figure S10 Mapping of the 5′ termini and frequency of the (−) GYSVd1-sRNAs from different tissues. Berry (A) tendril (B), flower (C) and leaf (D). Note that the scale is different in the panels and that 5′-3′ orientation is from right to left. Mapping is referred to GYSVd1 (−) genomic RNA (sequence variant GYSVd1.PN.22 with the accession number GQ995473).
(7.64 MB TIF)

Materials and Methods S1 Viroid-derived small RNAs (vd-sRNAs) in the sequenced libraries were retrieved searching for the 20–24 nt sRNAs perfectly matching the viroid sequence variants deposited in databases and with the accession number indicated below, and those identified in the present study.
(0.02 MB DOC)
