# Supplementary figures and images for: Deep Sequencing of Viroid-Derived Small RNAs from Grapevine Provides New Insights on the Role of RNA Silencing in Plant-Viroid Interaction
Source: PLoS One. 2009 Nov 5;4(11):e7686. doi: 10.1371/journal.pone.0007686 (PMC2767511; doi:10.1371/journal.pone.0007686)

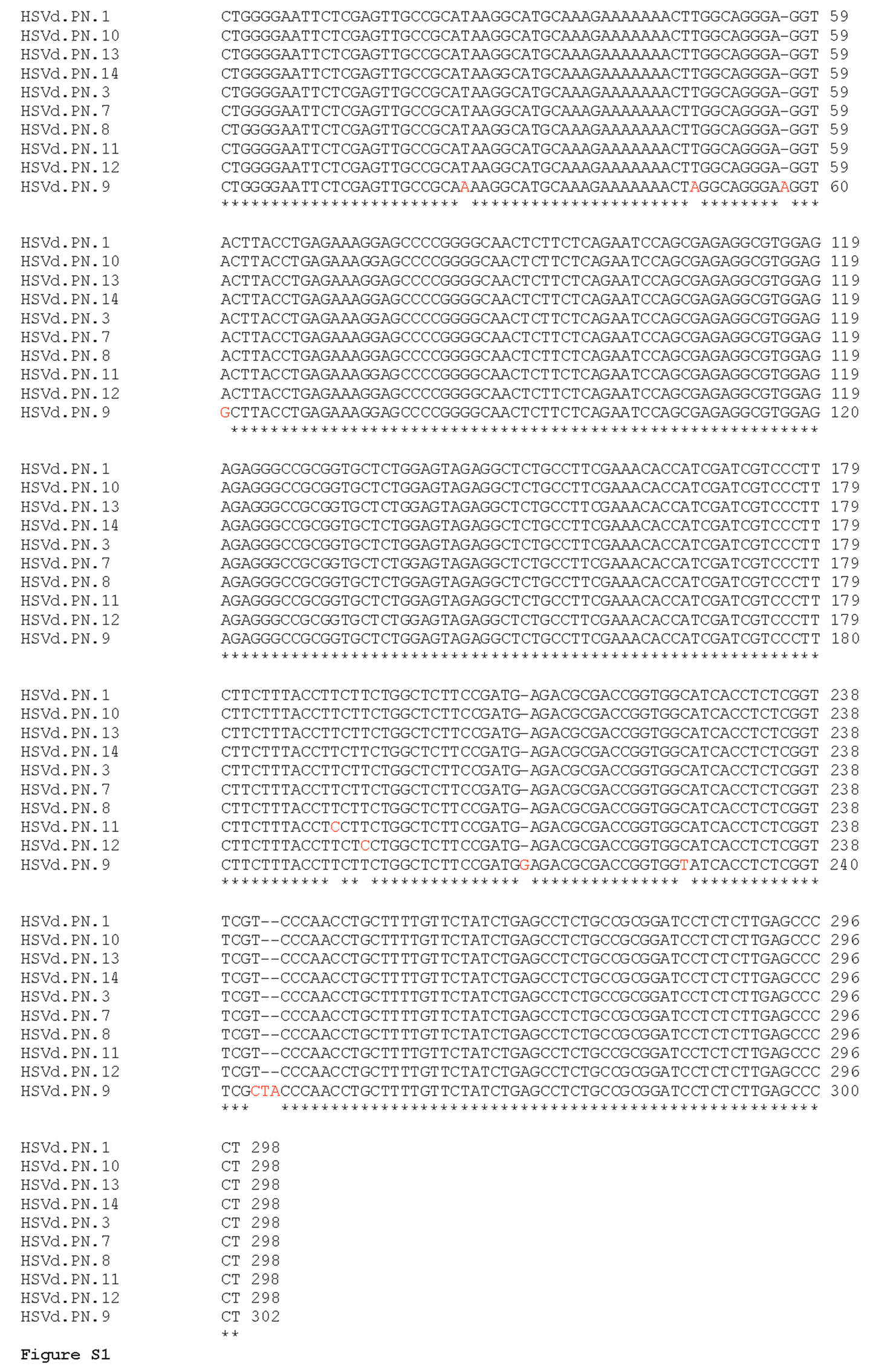

Supplement: Figure S1 — Multiple sequence alignment of HSVd cDNA variants from grapevine Pinot noir ENTAV115 identified in this study. Dashes and stars denote gaps and nucleotide identity, respectively. HSVd variants PN.1, PN.3, PN.7, PN.8, PN.10, PN.13 and PN.14 are identical to HSVd variant with accession number X06873, which is the master sequence in the infecting viroid population. Accession numbers for variants PN.9, PN.11 and PN.12 are GQ995464, GQ995465 and GQ995466, respectively. Nucleotides in red correspond to changes with respect to the master sequence. Numbers at the end of each line indicate nucleotide positions of each variant in the multiple alignment. (1.69 MB TIF) [file pone.0007686.s001.tif]

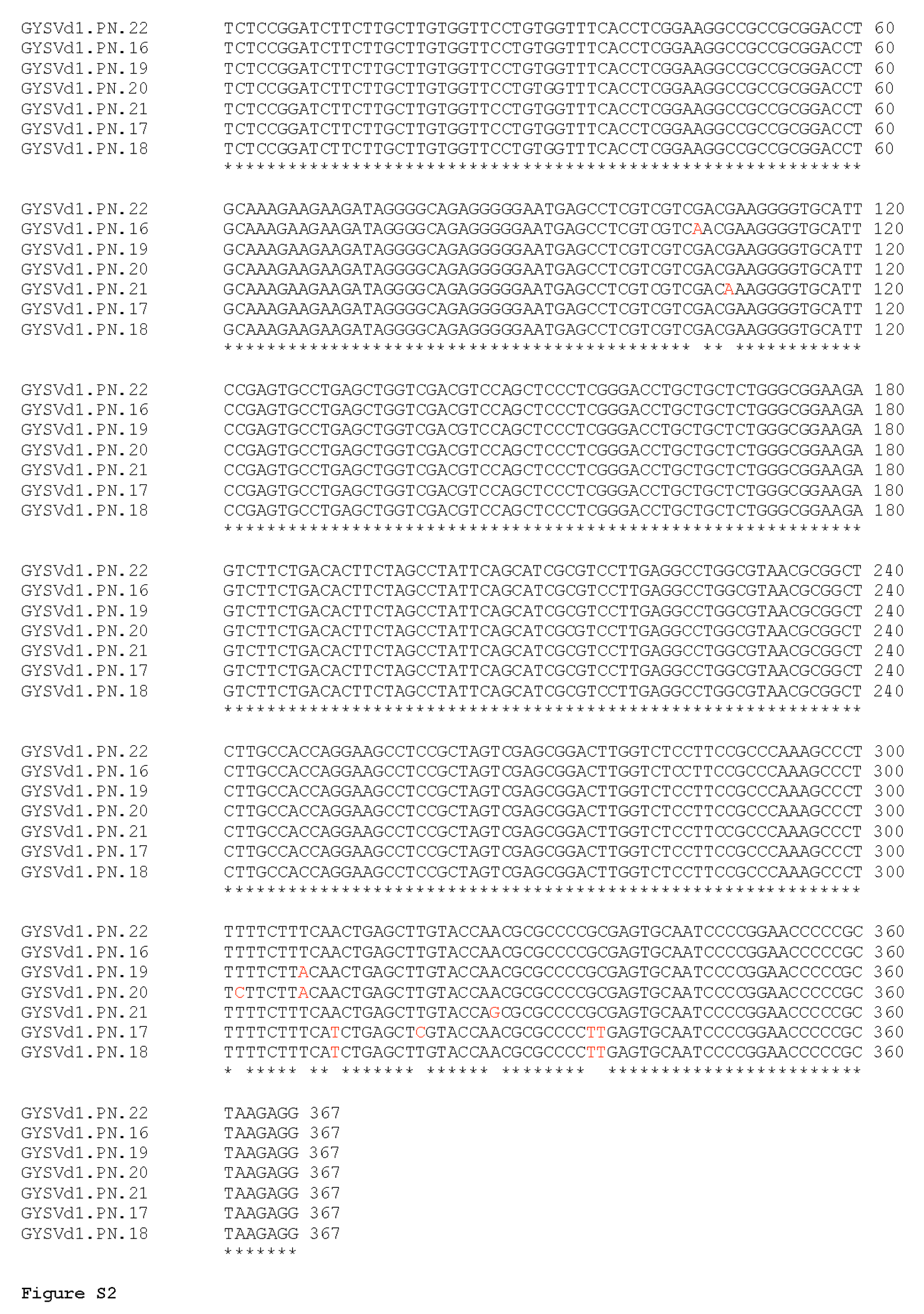

Supplement: Figure S2 — Multiple sequence alignment of GYSVd1 cDNA variants from grapevine Pinot noir clone ENTAV115 identified in the present study. Dashes and stars denote gaps and nucleotide identity, respectively. Nucleotides in red correspond to changes with respect to the consensus sequence, which corresponds to the sequence variant GYSVd1.PN.22 (accession number GQ995473) in the alignment. Numbers at the end of each line indicate nucleotide positions of each variant in the multiple alignment. (1.16 MB TIF) [file pone.0007686.s002.tif]

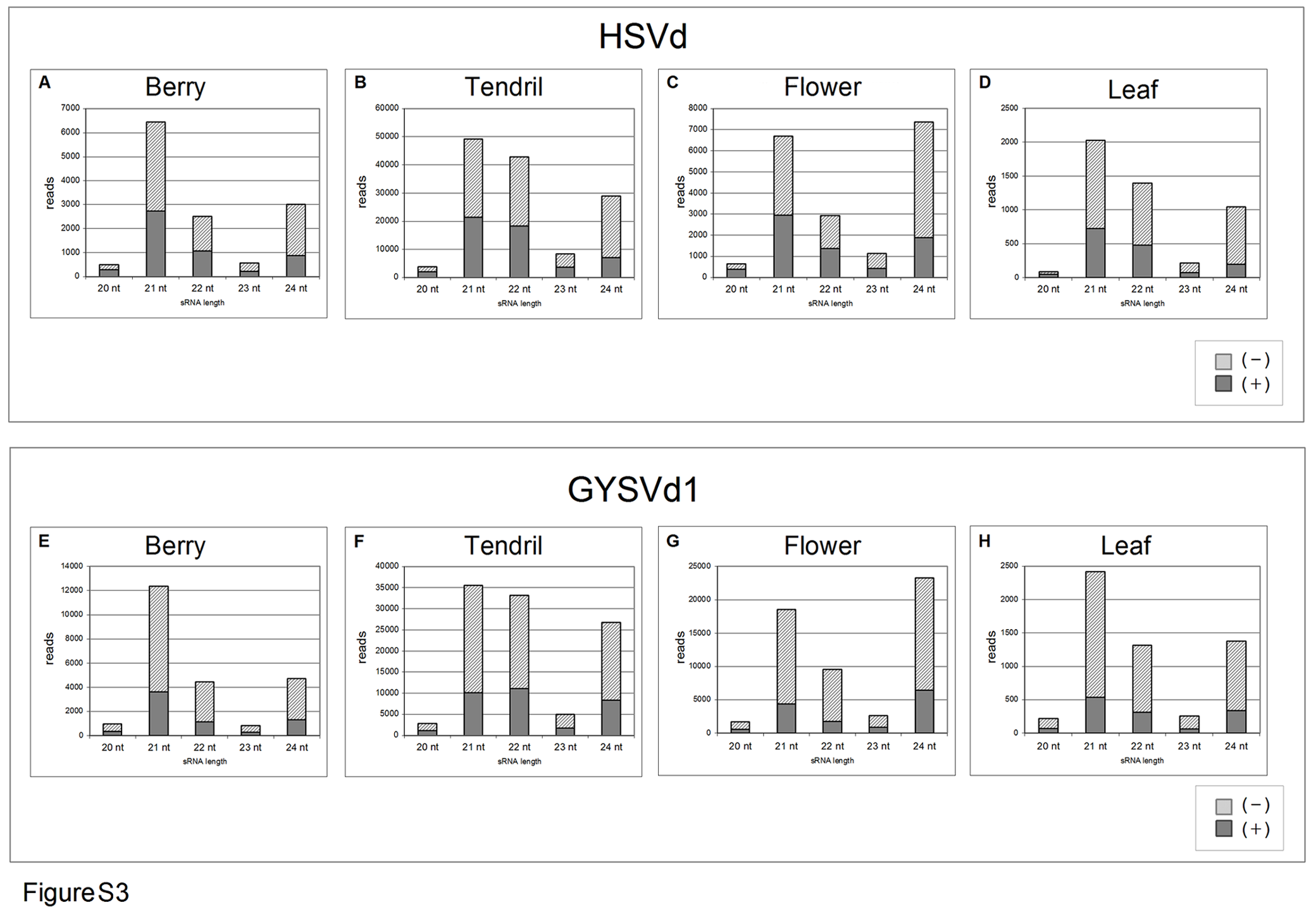

Supplement: Figure S3 — Size distribution (20–24 nt) of vd-sRNAs from different grapevine tissues. Histograms comparing the size distribution (20–24 nt) of HSVd- (upper panels) and GYSVd1-sRNAs (lower panels) isolated from berry (A and E), tendril (B and F), flower (C and G) and leaf (D and H). Vd-sRNAs of 21 nt of both viroids were the most abundant in berry, tendril and leaf samples, whereas the most prominent peak from the flower samples corresponded to 24-nt HSVd-sRNAs and GYSVd1-sRNAs. (8.98 MB TIF) [file pone.0007686.s003.tif]

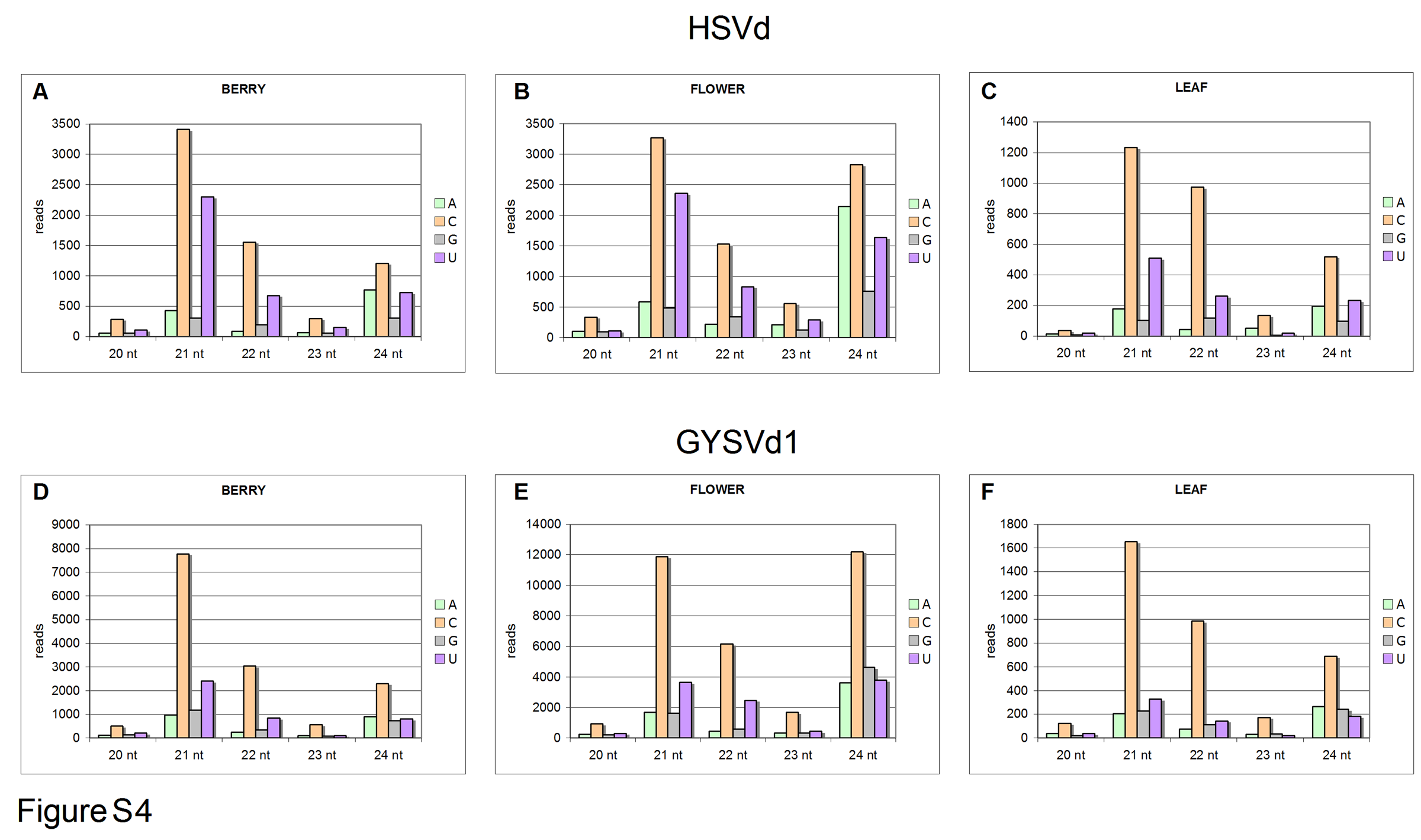

Supplement: Figure S4 — Frequency of the 5′-terminal nucleotide in vd-sRNAs. Histograms comparing the size distribution (20–24-nt) and nucleotide at the 5′ termini of HSVd-sRNAs (upper panels) and GYSVd1-sRNAs (lower panels) from berry (A and D), flower (B and E) and leaf (C and F). (10.22 MB TIF) [file pone.0007686.s004.tif]

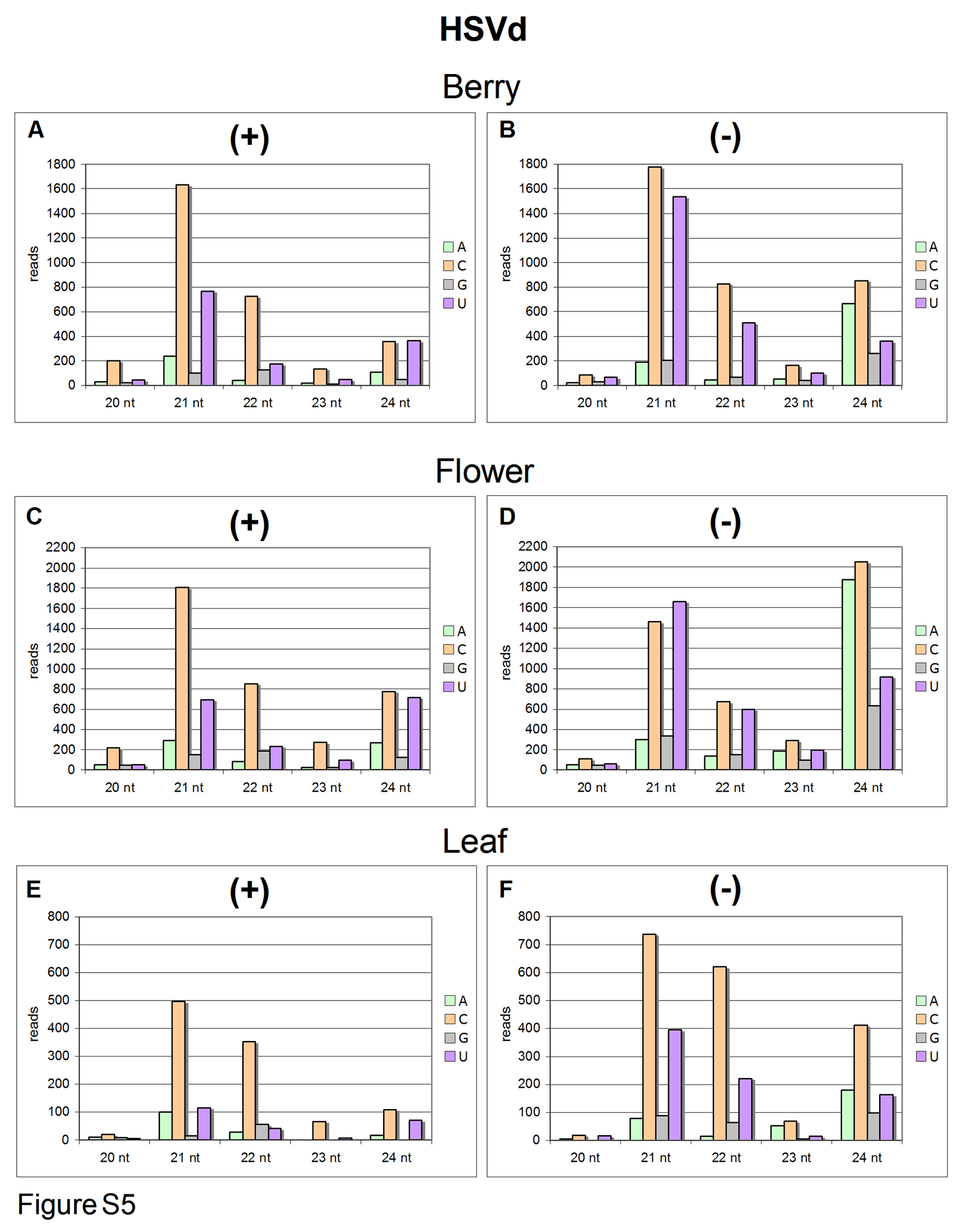

Supplement: Figure S5 — Frequency of the 5′-terminal nucleotide in (+) and (−) HSVd-sRNAs. Histograms comparing the size distribution (20–24-nt) and nucleotide at 5′ termini of (+) (left panels) and (−) (right panels) HSVd-sRNAs from berry (A and B), flower (C and D) and leaf (E and F). (9.92 MB TIF) [file pone.0007686.s005.tif]

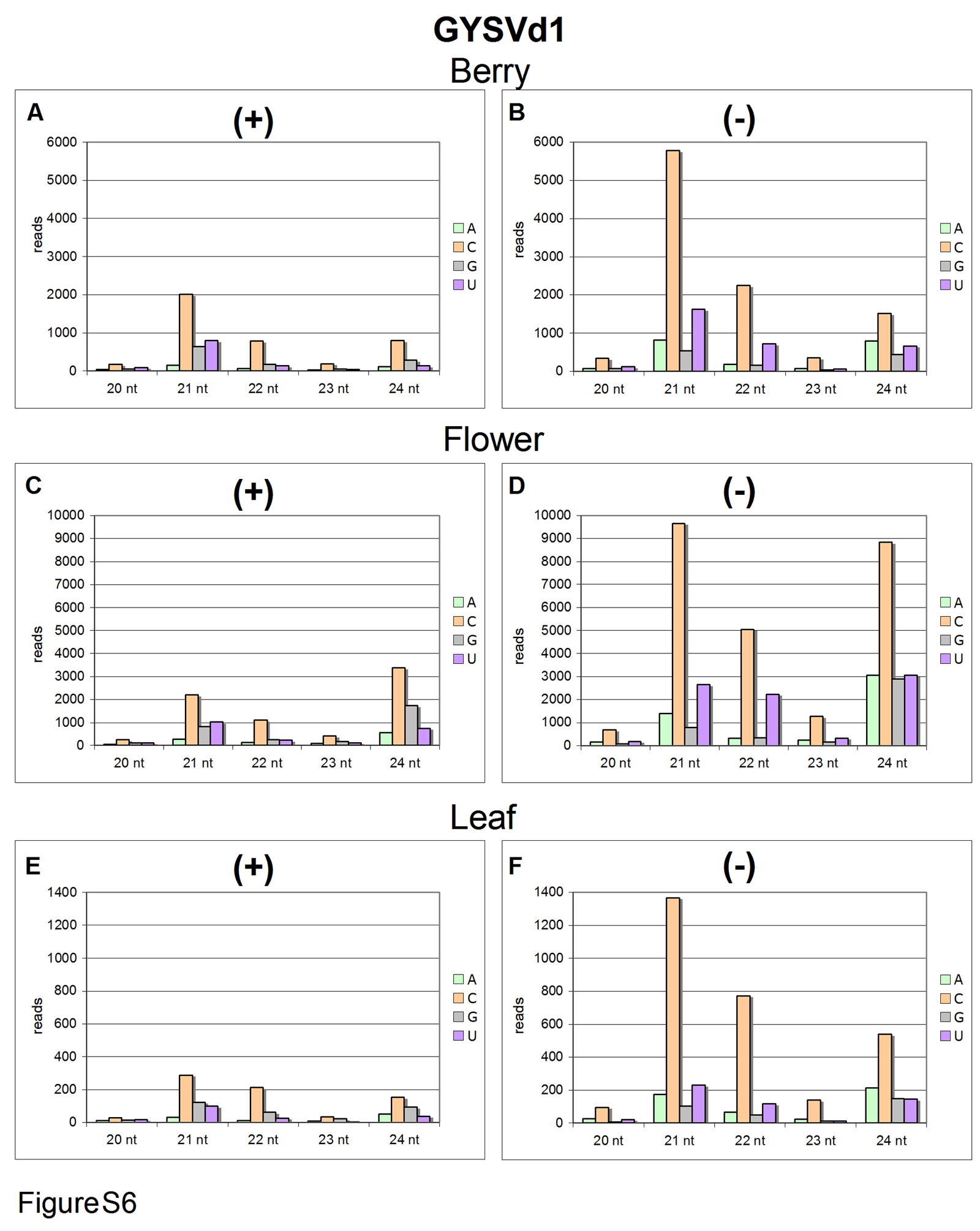

Supplement: Figure S6 — Frequency of the 5′-terminal nucleotide in (+) and (−) GYSVd1-sRNAs. Histograms comparing the size distribution (20–24-nt) and nucleotide at 5′ termini of (+) (left panels) and (−) (right panels) GYSVd1-sRNAs from berry (A and B), flower (C and D) and leaf (E and F). (9.65 MB TIF) [file pone.0007686.s006.tif]

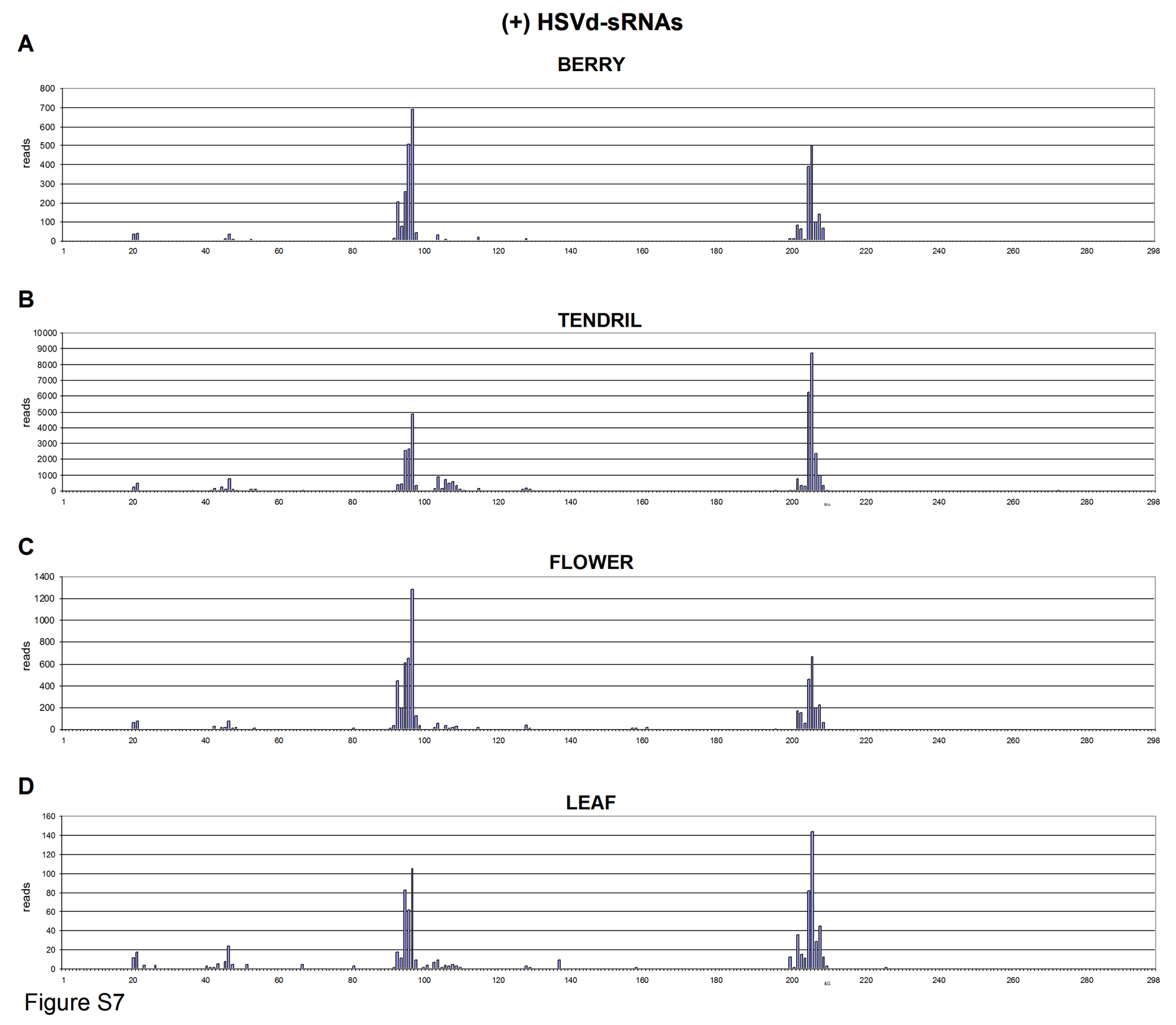

Supplement: Figure S7 — Mapping of the 5′ termini and frequency of (+) HSVd-sRNAs from different tissues. Berry (A), tendril (B), flower (C) and leaf (D). Note that the scale is different in the four panels and that 5′-3′ orientation is from left to right. Mapping is referred to the HSVd (+) genomic RNA (sequence variant with the accession number X06873). (9.65 MB TIF) [file pone.0007686.s007.tif]

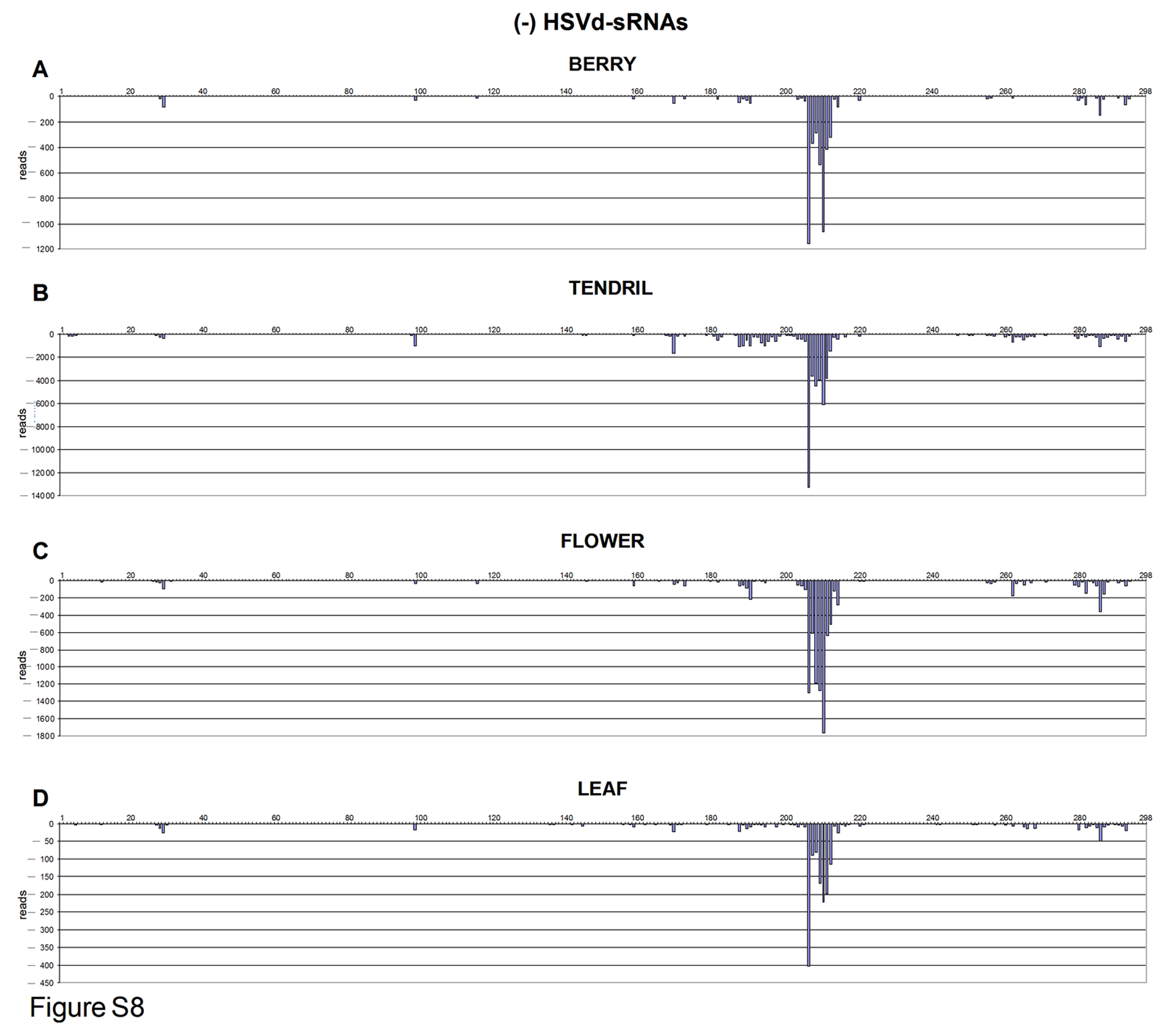

Supplement: Figure S8 — Mapping of the 5′ termini and frequency of the (−) HSVd-sRNAs from different tissues. Berry (A) tendril (B), flower (C) and leaf (D). Note that the scale is different in the four panels and that 5′-3′ orientation is from right to left. Mapping is referred to the HSVd (−) genomic RNA (sequence variant with the accession number X06873). (9.71 MB TIF) [file pone.0007686.s008.tif]

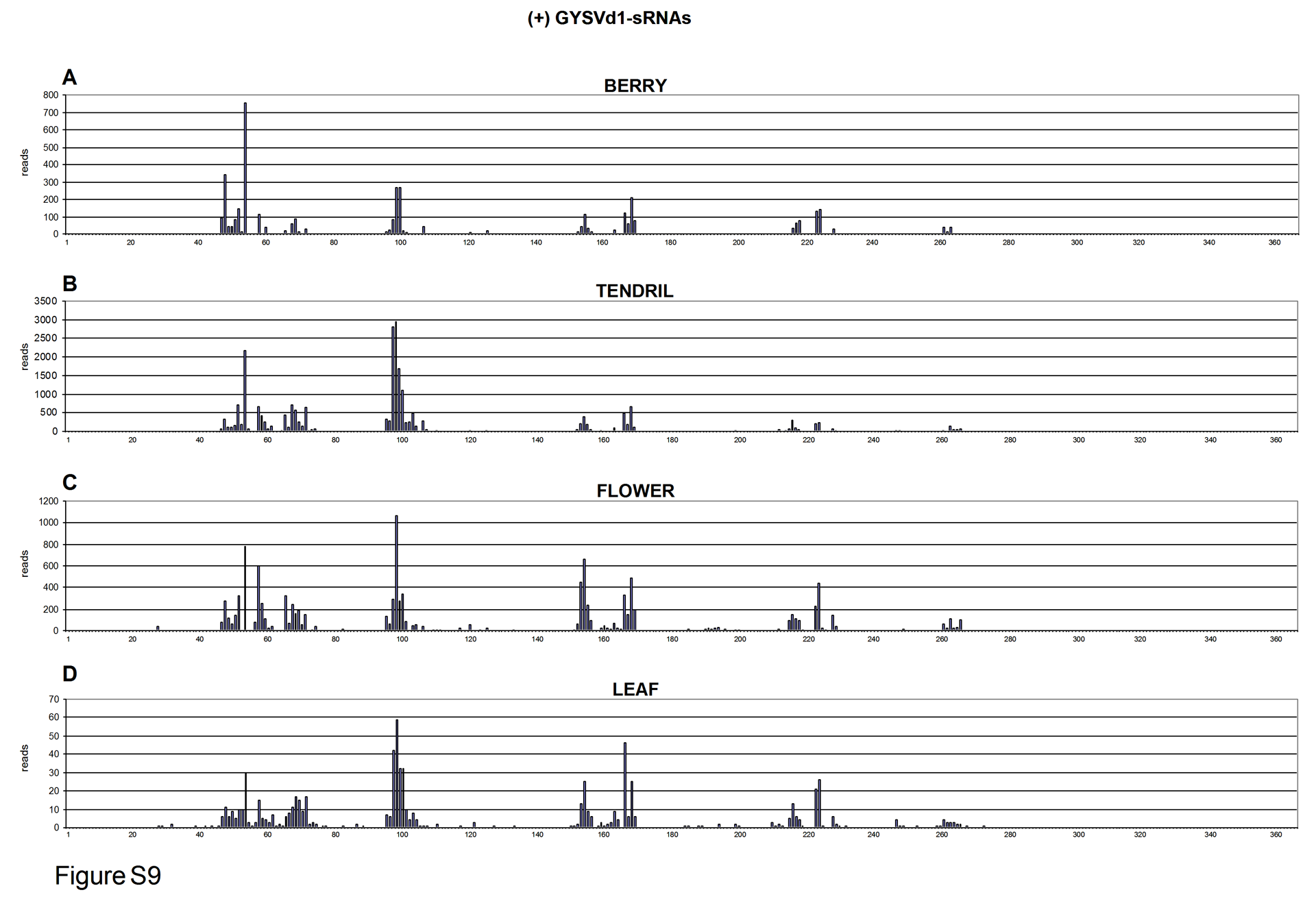

Supplement: Figure S9 — Mapping of the 5′ termini and frequency of the (+) GYSVd1-sRNAs from different tissues. Berry (A) tendril (B), flower (C) and leaf (D). Note that the scale is different in the four panels and that 5′-3′ orientation is from left to right. Mapping is referred to the GYSVd1 (+) genomic RNA (sequence variant GYSVd1.PN.22 with the accession number GQ995473). (7.56 MB TIF) [file pone.0007686.s009.tif]

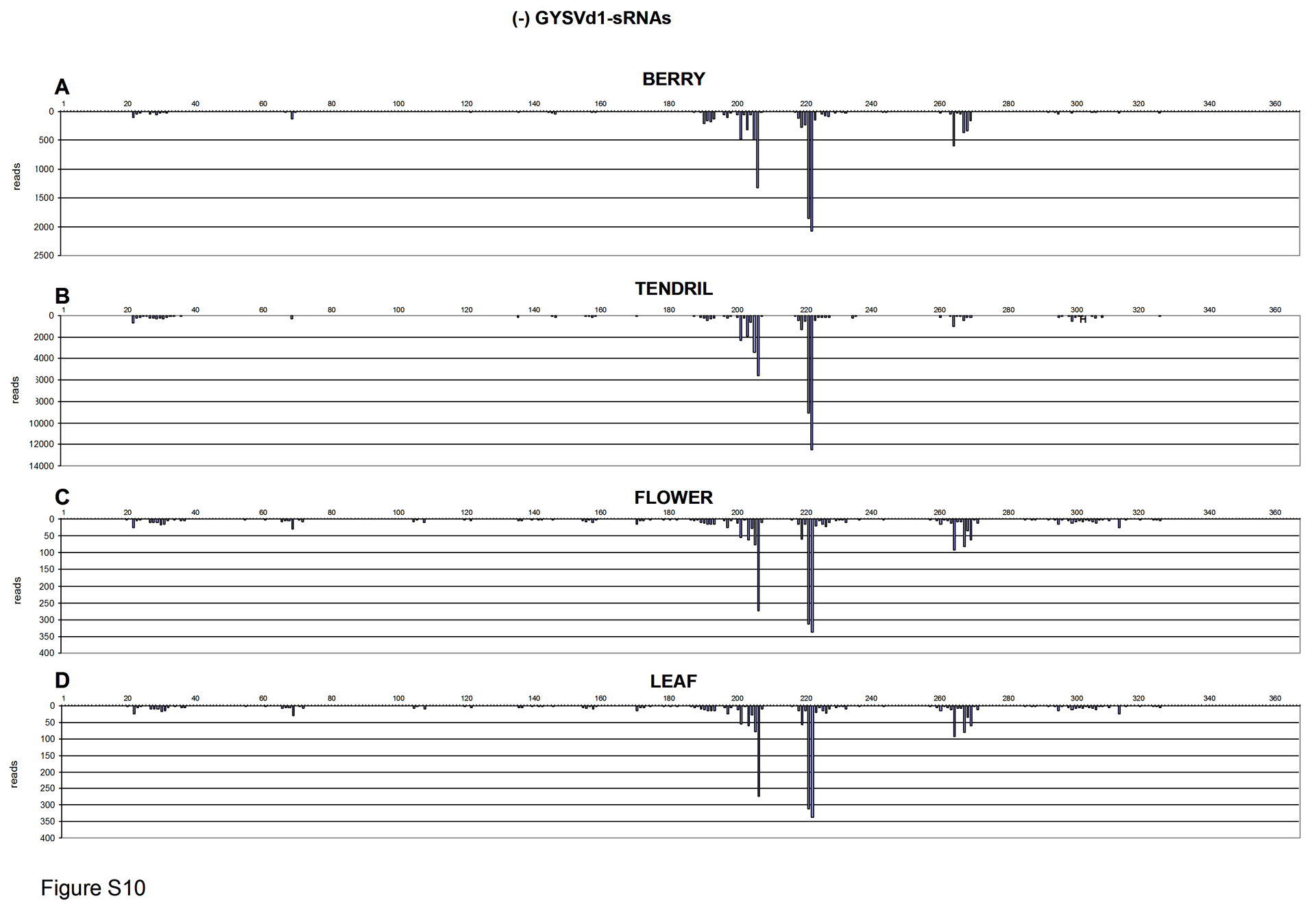

Supplement: Figure S10 — Mapping of the 5′ termini and frequency of the (−) GYSVd1-sRNAs from different tissues. Berry (A) tendril (B), flower (C) and leaf (D). Note that the scale is different in the panels and that 5′-3′ orientation is from right to left. Mapping is referred to GYSVd1 (−) genomic RNA (sequence variant GYSVd1.PN.22 with the accession number GQ995473). (7.64 MB TIF) [file pone.0007686.s010.tif]
