# Supplementary material for: Deep Sequencing of Viroid-Derived Small RNAs from Grapevine Provides New Insights on the Role of RNA Silencing in Plant-Viroid Interaction
Source: PLoS One. 2009 Nov 5;4(11):e7686. doi: 10.1371/journal.pone.0007686 (PMC2767511; doi:10.1371/journal.pone.0007686)
Supplement: Materials and Methods S1 — Viroid-derived small RNAs (vd-sRNAs) in the sequenced libraries were retrieved searching for the 20–24 nt sRNAs perfectly matching the viroid sequence variants deposited in databases and with the accession number indicated below, and those identified in the present study. (0.02 MB DOC) [file pone.0007686.s011.doc]

**Supporting information**

**Supplementary Materials and Methods S1**

Viroid-derived small RNAs (vd-sRNAs) in the sequenced libraries were retrieved searching for the 20-24 nt sRNAs perfectly matching the viroid sequence variants deposited in databases and with the accession number indicated below, and those identified in the present study (in bold characters and with the respective variant names in brackets) (see also Fig. S1 and S2).

**GYSVd1:**

NC_001920.1, Z17225.1, X87917.1, X87913.1, X87912.1, X87922.1, X87921.1, X87920.1, X87919.1, X87918.1, X87916.1, X87915.1, X87914.1, X87911.1, X87910.1, X87909.1, X87908.1, X87907.1, X87906.1, X87905.1, EU682454.1, EU682453.1, EU682452.1, DQ371477.1, DQ371476.1, DQ371475.1, DQ371474.1, DQ371473.1, DQ371472.1, DQ371471.1, DQ371470.1, DQ371469.1, DQ371467.1, DQ371468.1, DQ371466.1, DQ371465.1, DQ371464.1, DQ371463.1, DQ371462.1, DQ408542.1, AY639607.1, AY639606.1, AF462167.1, AF462166.1, AF462165.1, AF462164.1, AF462163.1, AF462162.1, AF462161.1, AF462160.1, AF462159.1, AF462158.1, AF462157.1, AF059712.1, AB028466.1, AB028465.1, **GQ995467 (GYSVd1.PN.16), GQ995468 (GYSVd1.PN.17), GQ995469 (GYSVd1.PN.18), GQ995470 (GYSVd1.PN.19), GQ995471 (GYSVd1.PN.20), GQ995472 (GYSVd1.PN.21), GQ995473 (GYSVd1.PN.22).**

**HSVd:**

NC_001351, Y14050.1, X06873.1, X87928.1, X87927.1, X87926.1, X87925.1, X87923.1, X87924.1, M35717.1,  **GQ995464 (HSV.PN.9), GQ995465 (HSVd.PN.11), GQ995466 (HSVd.PN.12)**
